# Effect of Adding Medium-Chain Triglyceride (MCT) Oil to Rice on Postprandial Glucose Response in Healthy Adults: A Pragmatic Within-Subject Trial

**DOI:** 10.7759/cureus.107143

**Published:** 2026-04-16

**Authors:** Kosuke Kodama, Masayasu Yoneda, Atsuko Kayashita, Jun Kayashita

**Affiliations:** 1 Graduate School of Comprehensive Scientific Research, Prefectural University of Hiroshima, Hiroshima, JPN; 2 Department of Diabetology and Internal Medicine, Shobara Red Cross Hospital, Hiroshima, JPN; 3 Faculty of Human Health Sciences, Meio University, Nago, JPN; 4 Faculty of Nutrition, Kobe Gakuin University, Kobe, JPN

**Keywords:** continuous glucose monitoring, glp-1, glycemic index, ketone bodies, medium-chain triglycerides, rice

## Abstract

Background and objectives: Postprandial hyperglycemia is a modifiable determinant of type 2 diabetes risk and vascular complications. Japonica rice produces pronounced postprandial glucose excursions. Medium-chain triglycerides (MCTs), absorbed via the portal vein, are rapidly oxidized in the liver and can stimulate glucagon-like peptide-1 (GLP-1) secretion and increase ketone body production, mechanisms that may attenuate postprandial glycemia.

Methods and study design: This study aimed to determine whether mixing MCT oil into cooked rice reduces postprandial interstitial glucose responses in healthy adults under real-world consumption conditions. In a within-subject, two-condition meal test, 32 participants consumed on separate mornings either a rice-only meal (150 g packed Japonica rice plus 8 g fish miso paste) or the same meal with 12 g MCT oil mixed immediately before intake. Available carbohydrate was standardized at 56.8 g in both meals. Interstitial glucose was measured with Freestyle Libre (Abbott Laboratories, Abbott Park, IL, USA) at 15-minute intervals for 120 minutes.

Results: The primary outcome was incremental area under the curve (iAUC₀-₁₂₀). Secondary outcomes included time-point glucose, subjective satiety (100-mm Visual Analog Scale (VAS)), and palatability (5-point Likert scale). Compared with the rice-only meal, the MCT meal significantly reduced interstitial glucose concentrations at 45, 60, and 75 minutes and lowered iAUC₀-₆₀, iAUC₀-₉₀, and iAUC₀-₁₂₀. Palatability ratings were lower for the MCT meal, whereas satiety did not differ between conditions.

Conclusions: Adding 12 g of MCT oil to 150 g of cooked rice attenuated postprandial glycemia without diminishing satiety. This simple, low-burden strategy may have practical utility for dietary glycemic management in everyday settings.

## Introduction

Postprandial glycemia contributes substantially to overall glycemic burden in individuals with early dysglycemia and type 2 diabetes [1-3]. Glucose elevations following carbohydrate-rich meals are associated with increased cardiometabolic risk and can be ameliorated through targeted dietary interventions [4,5]. The glycemic index (GI) and glycemic load frameworks are widely used to assess carbohydrate quality, and white rice consistently falls within the high-GI category [6,7]. Japanese short-grain rice, in particular, typically exhibits a GI in the upper 70s, reflecting its rapid digestion and absorption kinetics [7]. Consequently, an intervention that can be applied directly to rice, the primary carbohydrate source in Japan, may offer a practical means of improving postprandial glycemic control without necessitating major changes in meal structure. Medium-chain triglycerides (MCTs) enter the portal circulation directly and are rapidly oxidized in the liver, leading to increased ketone body production and potential modulation of glucose homeostasis [8]. In addition, MCTs have been shown to stimulate glucagon-like peptide-1 (GLP-1) secretion and slow gastric emptying, mechanisms that together may blunt postprandial glucose excursions by enhancing glucose-dependent insulinotropic effects and reducing the rate of nutrient delivery to the small intestine [9-11]. Because MCT oil is unsuitable for high-temperature cooking, the most practical method for incorporating MCTs into daily meals is to mix a measured amount into freshly heated rice immediately before consumption [12]. This approach is simple, feasible in both home and institutional kitchens, and consistent with common preparation methods for packaged rice in Japan. The present study examined this real-world application in healthy young adults. Flash continuous glucose monitoring (CGM) was used to quantify interstitial glucose responses, and acceptability was evaluated using palatability scores and subjective satiety assessments.

## Materials and methods

Study design and participants

This study employed a pragmatic within-subject design to minimize between-person variability and to approximate everyday meal-consumption behaviors rather than strictly controlled laboratory conditions. All procedures were conducted in accordance with the Declaration of Helsinki and were approved by the Ethics Committee of the Prefectural University of Hiroshima, Hiroshima, Japan (Approval No. 23HH016). Written informed consent was obtained from all participants prior to participation. A total of 36 university students were initially recruited. Exclusion criteria included age under 18 years, a body mass index (BMI) ≥25 kg/m², pregnancy or lactation, a history of gastrointestinal surgery, known impaired glucose tolerance, clearly impaired cognition, or food allergies relevant to the test meals. Four participants did not complete both test conditions due to unrelated illness, measurement error, or protocol deviations. Consequently, 32 participants (eight males and 24 females; mean age 21.2 ± 0.7 years; BMI 20.3 ± 1.7 kg/m²) were included in the final analysis.

Test meals

Each participant consumed two breakfast meals on separate mornings. The control meal consisted of 150 g of heated packaged Japonica rice accompanied by 8 g of fish miso paste. The intervention meal was identical except that 12 g of commercially available MCT oil was thoroughly mixed into the rice immediately before consumption. Heating was performed according to the manufacturer’s instructions. Available carbohydrate was standardized at 56.8 g for both meals (Table 1). The study was conducted in two distinct periods: the first from February 8 to 20, 2024, and the second from May 9 to 23, 2024. Since the Freestyle Libre (Abbott Laboratories, Abbott Park, IL, USA) was attached on Day 1, participants maintained their habitual diet without test meals on Days 1 and 2. Administration of the test meals commenced on Day 3. Participants were instructed to avoid strenuous exercise, overeating, and alcohol consumption on the day preceding the test, to fast for at least eight hours before each test meal, and to consume each meal within 10 minutes. During the subsequent two-hour postprandial period, participants remained seated indoors and were permitted to consume only water.

**Table 1:** Nutritional composition of test meals MCT: medium-chain triglyceride

Component	Rice only	Rice+MCT
Energy (kcal)	257	365
Protein (g)	3.5	3.5
Fat (g)	0.4	12.4
Available carbohydrate (g)	56.8	56.8
Dietary fiber (g)	2.25	2.25
Salt equivalent (g)	0.4	0.4

Glucose measurement

Interstitial glucose concentrations were measured using the factory-calibrated flash CGM system (Freestyle Libre). Sensors were placed on the upper arm contralateral to the dominant hand. Because CGM accuracy is reduced on the first day of wear, all tests were conducted from the second day onward [13]. Glucose values were recorded at baseline and at 15-minute intervals for 120 minutes after meal ingestion. Although interstitial glucose measurements lag slightly behind capillary glucose concentrations, CGM-derived values are considered acceptable for evaluating postprandial glycemic responses.

Outcome measures

The primary outcome was the incremental area under the curve from 0 to 120 minutes (iAUC₀-₁₂₀), calculated relative to baseline. Secondary outcomes included iAUC₀-₆₀, iAUC₀-₉₀, time-point interstitial glucose concentrations, subjective satiety assessed using a 100-mm Visual Analog Scale (VAS) at 0, 30, 60, and 120 minutes, and palatability evaluated using a 5-point Likert scale.

Statistical analysis

Statistical analyses were performed within a within-subject framework. iAUC values were calculated using the trapezoidal rule relative to baseline. Continuous variables are presented as mean ± standard deviation (SD) unless otherwise specified. Time-point interstitial glucose concentrations and iAUC values were compared between conditions using paired t-tests. To account for multiplicity, formal statistical inference focused on the prespecified primary endpoint (iAUC₀-₁₂₀), whereas analyses of secondary endpoints were considered exploratory. To explore potential sex-related effect modification, linear mixed-effects models were constructed with iAUC or time-point glucose concentrations as dependent variables. Meal condition, sex, and the meal-condition-by-sex interaction were included as fixed effects, and participant ID was included as a random effect. The interaction term evaluated sex-specific differences in response.

## Results

**Figure 1:**
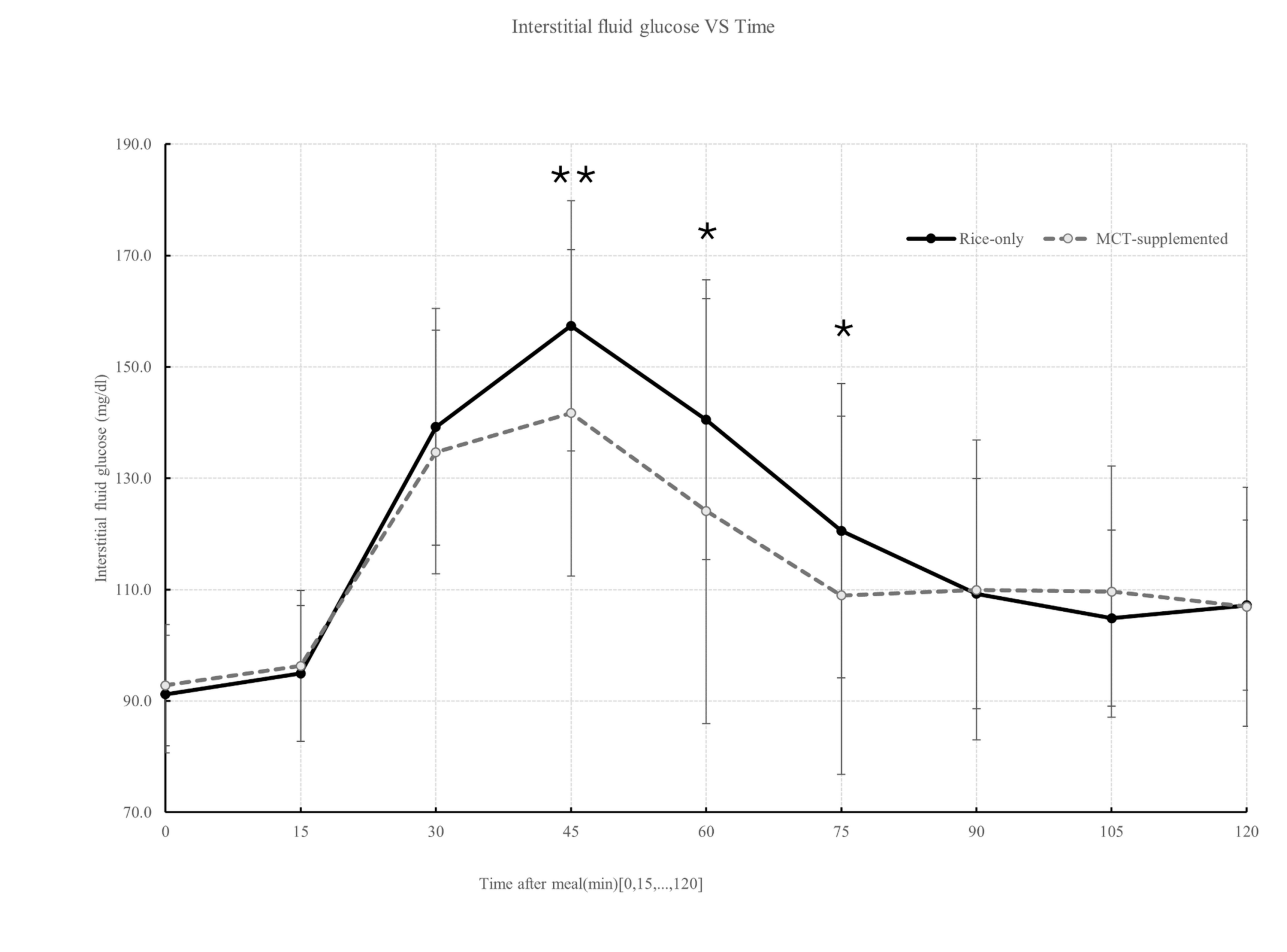
Postprandial interstitial glucose profiles after rice intake with or without MCT oil Interstitial fluid glucose concentration over 120 minutes after rice-only and MCT-supplemented meals (n = 32). Values are mean ± SD. *p < 0.05; *p < 0.01 versus rice-only meal (paired t-test). MCT: medium-chain triglyceride

**Figure 2:**
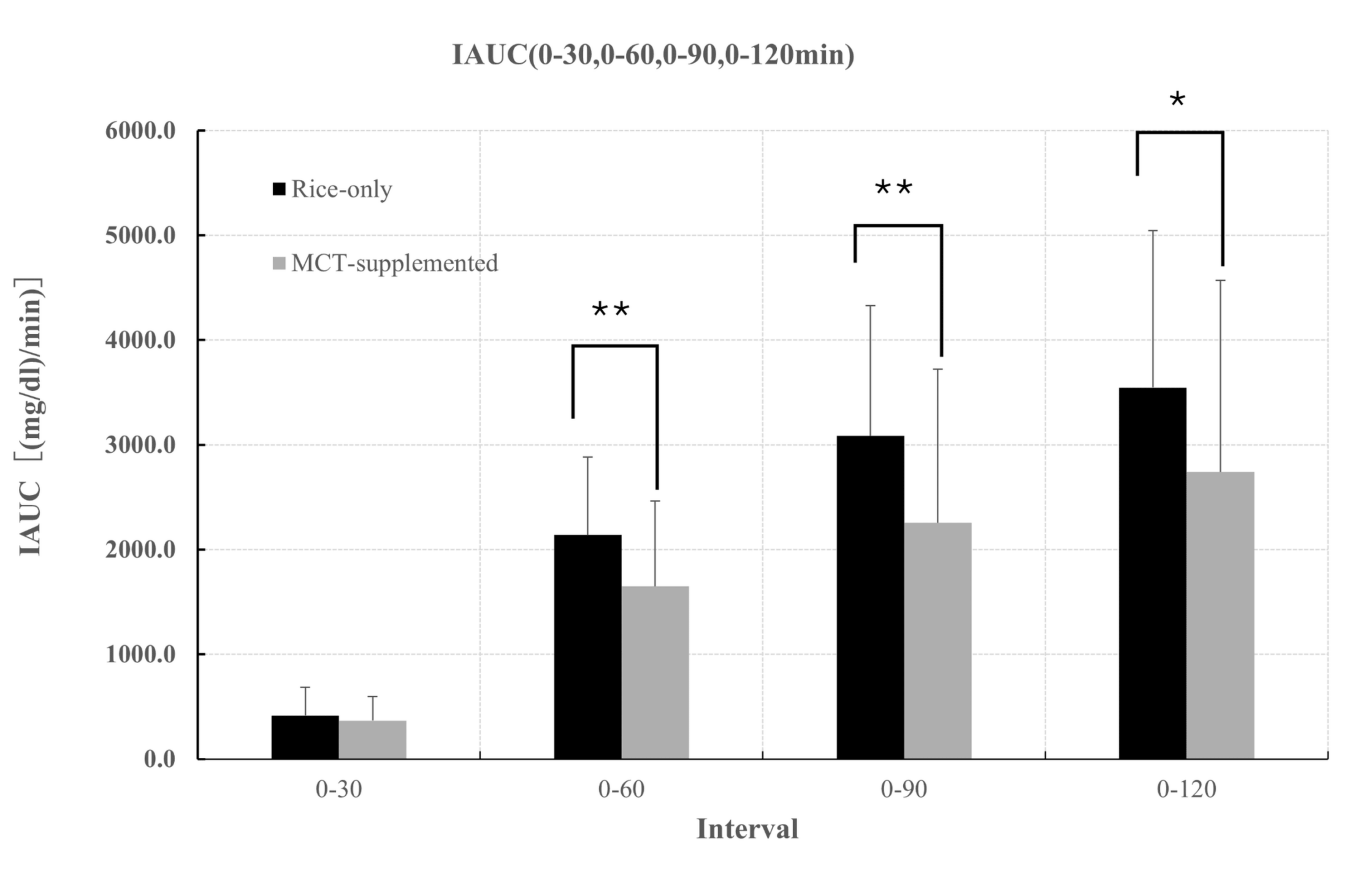
Incremental area under the curve (iAUC) of postprandial interstitial glucose after rice intake with or without MCT oil iAUC for interstitial glucose over 0–30, 0–60, 0–90, and 0–120 min after rice-only and MCT-supplemented meals. Values are mean ± SD (n = 32). *p < 0.05, *p < 0.01 versus rice-only meal (paired t-test). MCT: medium-chain triglyceride

**Figure 3:**
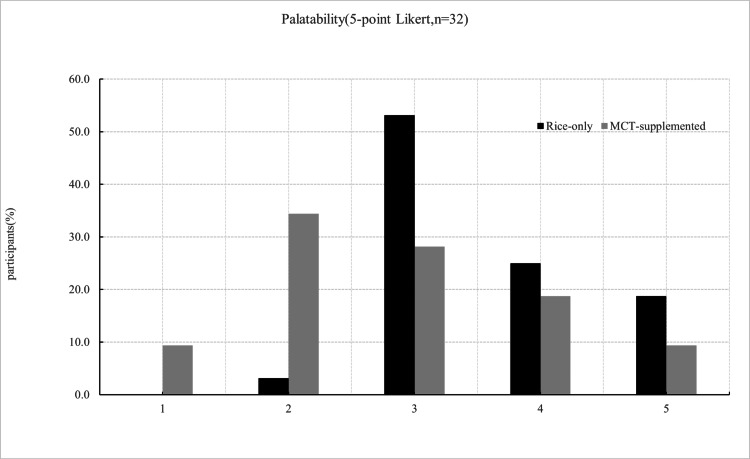
Distribution of palatability ratings for rice-only and MCT-supplemented rice using a 5-point Likert scale Distribution of palatability ratings (5-point Likert scale) for rice-only and MCT-supplemented meals (n = 32). Values represent the percentage of participants selecting each response category. The MCT meal showed significantly lower palatability ratings compared with the rice-only meal (p < 0.05, paired comparison). MCT: medium-chain triglyceride

**Figure 4:**
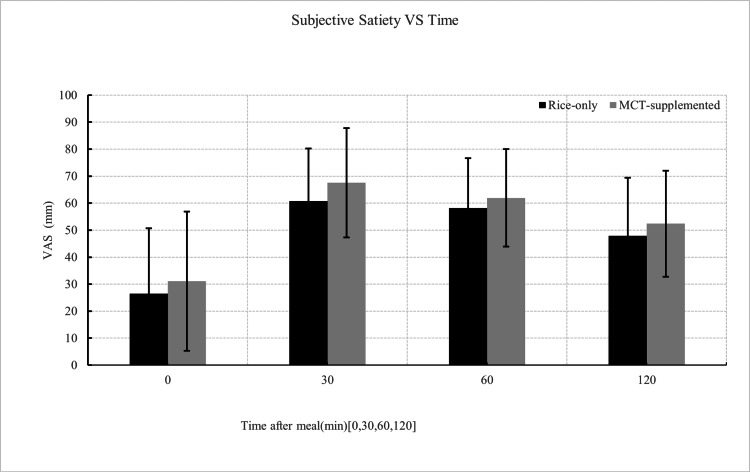
Postprandial changes in subjective satiety after Rice intake with or without MCT oil Subjective satiety (visual analogue scale (VAS)) after rice-only and MCT-supplemented meals (n = 32). Values are presented as mean ± SD. No significant differences were observed between meals at any time point (p > 0.05, paired comparison). MCT: medium-chain triglyceride

Of the 36 individuals initially enrolled, 32 participants completed both meal conditions and were included in the final analysis. Exclusions were due to unrelated illness, measurement errors, or protocol deviations. All included participants adhered to the study procedures, and no medically relevant adverse events were observed. Overall, the analytic cohort consisted of healthy, young, normal-weight adults without known metabolic disorders. Figure 1 illustrates the mean interstitial glucose trajectories following the rice-only and MCT-supplemented meals over the 120-minute postprandial period. Interstitial glucose concentrations were significantly lower following the MCT meal at 45, 60, and 75 minutes compared with the rice-only condition (45 min: t = -3.10, p = 0.002; 60 min: t = -1.95, p = 0.03; 75 min: t = -1.70, p = 0.049). Consistent with these time-point differences, iAUC analyses demonstrated significantly lower glycemic exposure following the MCT meal. iAUC₀-₆₀, iAUC₀-₉₀, and the primary endpoint iAUC₀-₁₂₀ were all significantly reduced compared with the rice-only condition (0-60: t = -2.95, p = 0.003; 0-90: t=-2.45, p=0.01; 0-120: t=-2.14, p=0.02; Figure 2). Palatability ratings differed between conditions, with the MCT-supplemented meal receiving lower scores on the 5-point Likert scale (Table 2; Figure 3). In contrast, subjective satiety scores assessed using the VAS did not differ significantly between conditions at any time point (Figure 4). Exploratory mixed-effects analyses examining sex-related differences showed that the direction of effect was consistent in both males and females, with lower iAUC values following the MCT meal. However, the meal-condition-by-sex interaction was not statistically significant (p = 0.43; Table 3). Descriptive within-sex mean differences and corresponding 95% confidence intervals are summarized in Tables 4-7.

**Table 2:** Palatability scores (five‑point Likert) by condition (n = 32). MCT: medium-chain triglyceride

Score	Rice only n (%)	Rice+MCT n (%)
5 (Very good)	6 (18.8%)	3 (9.4%)
4 (Good)	8 (25.0%)	6 (18.8%)
3 (Neutral)	17 (53.1%)	9 (28.1%)
2 (Bad)	1 (3.1%)	11 (34.4%)
1 (Very bad)	0 (0.0%)	3 (9.4%)

**Table 3:** Mixed-effects model analysis of incremental area under the curve (iAUC) across time intervals (0–30, 0–60, 0–90, and 0–120 minutes)

Time (in minutes)	Interaction effect
	(Condition×Gender)
0-30	0.7
0-60	0.748
0-90	0.661
0-120	0.425

**Table 4:** Mean differences in postprandial interstitial glucose concentrations between rice-only and rice+MCT meals in female participants (n = 24) MCT: medium-chain triglyceride

Time (in minutes)	Mean (mg/dL)	SD	SE	95% CI Lower	95% CI Upper
0	1.0	10.9	0.5	0.0	23.5
15	1.0	12.8	0.5	-0.1	27.4
30	-5.2	23.5	1.0	-7.2	43.4
45	-11.1	26.0	1.1	-13.4	42.7
60	-10.6	40.0	1.7	-14.1	72.1
75	-6.5	30.4	1.3	-9.1	56.5
90	0.0	24.1	1.0	-2.1	49.8
105	4.1	22.9	1.0	2.2	51.4
120	0.0	21.8	0.9	-1.9	45.1

**Table 5:** Mean differences in postprandial interstitial glucose concentrations between rice-only and rice+MCT meals in male participants (n = 8) MCT: medium-chain triglyceride

Time (in minutes)	Mean (mg/dL)	SD	SE	95% CI Lower	95% CI Upper
0	3.6	12.2	1.5	0.0	7.2
15	2.5	16.0	2.0	-2.2	7.2
30	-2.5	17.5	2.2	-7.7	2.7
45	-29.3	19.2	2.4	-34.9	-23.6
60	-33.6	39.3	4.9	-45.2	-22.0
75	-27.0	33.6	4.2	-36.9	-17.1
90	2.8	18.3	2.3	-2.7	8.2
105	6.6	14.2	1.8	2.4	10.8
120	-1.0	14.7	1.8	-5.3	3.3

**Table 6:** Differences in incremental area under the curve (iAUC) for interstitial glucose between rice-only and rice+MCT meals in female participants (n = 24) MCT: medium-chain triglyceride

Time (in minutes)	Mean (mg·min/dL)	SD	SE	95% CI Lower	95% CI Upper
0-30	-46.3	307.0	12.8	-72.7	588.9
0-60	-360.6	913.4	38.1	-439.4	1528.9
0-90	-565.9	1596.4	66.5	-703.5	2736.5
0-120	-533.1	1959.7	81.7	-702.0	3520.9

**Table 7:** Differences in incremental area under the curve (iAUC) for interstitial glucose between rice-only and rice+MCT meals in male participants (n = 8) MCT: medium-chain triglyceride

Time (in minutes)	Mean (mg·min/dL)	SD	SE	95% CI Lower	95% CI Upper
0-30	-62.8	253.1	31.6	-137.6	12.0
0-60	-881.3	659.0	82.4	-1076.0	-686.5
0-90	-1626.6	1275.2	159.4	-2003.5	-1249.6
0-120	-1622.8	1409.0	176.1	-2039.3	-1206.3

## Discussion

This pragmatic within-subject study demonstrated that incorporating 12 g of MCT oil into a 150 g rice meal significantly attenuated postprandial interstitial glucose excursions in healthy young adults. The attenuation was observed at several mid-postprandial time points and was supported by reductions across all iAUC intervals. Because the intervention relied solely on routine heating and mixing procedures identical to those used in everyday meal preparation, the findings support the practical feasibility of implementing this strategy in home, institutional, and community settings. Several physiological mechanisms may account for the observed reduction in glycemia. MCTs have been reported to stimulate GLP-1 secretion, enhance glucose-dependent insulin release, and slow gastric emptying, each contributing to reductions in postprandial glucose elevations for a fixed carbohydrate load [8-11]. Rapid hepatic β-oxidation of MCTs also increases ketone body availability, which may shift substrate utilization and modulate hepatic glucose output [8]. Furthermore, incorporating oil into the meal bolus may influence gastric sieving and slow the delivery of nutrients to the small intestine, thereby reducing the rate at which glucose enters the circulation [14]. Despite these metabolic advantages, reduced palatability emerged as a practical barrier. Participants frequently described altered mouthfeel and decreased adhesiveness, textural characteristics typically associated with Japanese short-grain rice, when MCT oil was mixed in rice [12]. To improve acceptability, several culinary strategies may be effective, such as dispersing smaller quantities of oil throughout the meal, combining MCT oil with condiments (e.g., nori, sesame), or emulsifying it into miso soup or other side dishes. These adaptations may help preserve both sensory qualities and metabolic benefits. From a public-health standpoint, directing an intervention toward rice, the predominant carbohydrate source in Japan and many Asian countries, is particularly attractive. High-GI rice contributes substantially to postprandial glycemic exposure [15,16], and modest improvements at each meal may accumulate into meaningful metabolic benefits among individuals who consume rice multiple times daily. Although rice consumption in Japan has gradually declined due to dietary westernization [17], national rice availability remains several-fold higher than in Western countries [1,18], making the proposed approach culturally compatible and easy to implement. The absence of satiety differences between meals also suggests potential utility in older adults who require higher energy intake to prevent frailty and sarcopenia while minimizing glycemic excursions [19-21]. Furthermore, MCT oil requires no specialized preparation, is low-cost, and can be readily implemented in home-care and institutional settings. Scaling this strategy would benefit from an implementation-science framework. Engagement of stakeholders, including patients, caregivers, dietitians, and food-service personnel, may facilitate recipe optimization, palatability improvements, and integration into routine workflows. Pre-measured MCT sachets could support standardized use in hospitals and long-term care facilities. Safety considerations include gastrointestinal tolerance and potential interactions with lipid-lowering medications [12,22]. Health economic evaluations may help determine cost-effectiveness relative to alternative dietary strategies for glycemic control. Several limitations should be acknowledged. The fixed meal order may have introduced sequence or time-of-day effects. The sample consisted of healthy young adults, limiting generalizability to older individuals or those with metabolic impairments. Because interstitial glucose lags behind capillary glucose, very early postprandial dynamics may have been underestimated. Additionally, mechanistic parameters, including GLP-1, ketone bodies, and gastric emptying, were not directly measured, and physiological interpretations remain inferential. Furthermore, regarding generalizability, although the present study focused exclusively on Japanese white rice, the results may vary depending on rice cultivar and cooking methods. Future research should therefore examine the effects in other rice varieties as well as in alternative staple foods such as corn. Future research should employ randomized crossover designs, evaluate multiple MCT doses, and include older adults and individuals with impaired glucose tolerance. Concurrent measurement of GLP-1, ketone bodies, and gastric emptying markers would clarify underlying physiological pathways. Studies focusing on texture modification, emulsification, and qualitative sensory evaluation may enhance palatability while maintaining metabolic efficacy. Combining MCT with dietary fiber or protein may also yield added glycemic benefits [5,23]. Standardized reporting of iAUC computation would facilitate comparisons across studies. Sex-related differences in gastric emptying and incretin responses have been reported previously [24,25], raising the possibility of sex-based effect modification. Although our exploratory mixed-effects analysis did not detect a statistically significant condition × sex interaction, the unbalanced sex distribution limits interpretation. Adequately powered trials stratified by sex are therefore warranted. Given the exploratory nature of this study, a post hoc power analysis was performed to evaluate the validity of the obtained results. For the interstitial fluid glucose level at 45 minutes and the iAUC₀₋₉₀ min, where the most prominent differences were observed, the statistical power was calculated based on a paired t-test (two-tailed, α = 0.05, n = 32). The analysis revealed an effect size of 1.41 and a power of 1.00 for both indices, confirming that the significant differences observed in this study have sufficient statistical reliability. In summary, this study demonstrates that mixing a modest amount of MCT oil into cooked rice is a feasible and physiologically effective strategy for reducing postprandial glycemia. Given its simplicity, cultural compatibility, and potential relevance for individuals at risk of frailty or metabolic dysregulation, this approach merits further evaluation in diverse real-world settings.

## Conclusions

In conclusion, adding a modest amount of MCT oil to cooked rice significantly attenuated postprandial interstitial glucose excursions without reducing subjective satiety. Because this intervention requires no specialized preparation and is compatible with habitual dietary practices in rice-eating cultures, it represents a practical and scalable approach to dietary glycemic management. Further studies involving older adults, individuals with impaired glucose tolerance, and patients with type 2 diabetes are needed to confirm the generalizability and long-term metabolic impact of this strategy.
